# Future challenges for vection research: definitions, functional significance, measures, and neural bases

**DOI:** 10.3389/fpsyg.2015.00193

**Published:** 2015-02-27

**Authors:** Stephen Palmisano, Robert S. Allison, Mark M. Schira, Robert J. Barry

**Affiliations:** ^1^School of Psychology, University of WollongongWollongong, NSW, Australia; ^2^Department of Electrical Engineering and Computer Science, York UniversityToronto, ON, Canada

**Keywords:** vection, self-motion perception, optic flow, egomotion, conscious experience, functional significance, neural basis

## Abstract

This paper discusses four major challenges facing modern vection research. Challenge 1 (Defining Vection) outlines the different ways that vection has been defined in the literature and discusses their theoretical and experimental ramifications. The term vection is most often used to refer to visual illusions of self-motion induced in stationary observers (by moving, or simulating the motion of, the surrounding environment). However, vection is increasingly being used to also refer to non-visual illusions of self-motion, visually mediated self-motion perceptions, and even general subjective experiences (i.e., “feelings”) of self-motion. The common thread in all of these definitions is the conscious subjective experience of self-motion. Thus, Challenge 2 (Significance of Vection) tackles the crucial issue of whether such conscious experiences actually serve functional roles during self-motion (e.g., in terms of controlling or guiding the self-motion). After more than 100 years of vection research there has been surprisingly little investigation into its functional significance. Challenge 3 (Vection Measures) discusses the difficulties with existing subjective self-report measures of vection (particularly in the context of contemporary research), and proposes several more objective measures of vection based on recent empirical findings. Finally, Challenge 4 (Neural Basis) reviews the recent neuroimaging literature examining the neural basis of vection and discusses the hurdles still facing these investigations.

## INTRODUCTION

**FIGURE 1:**
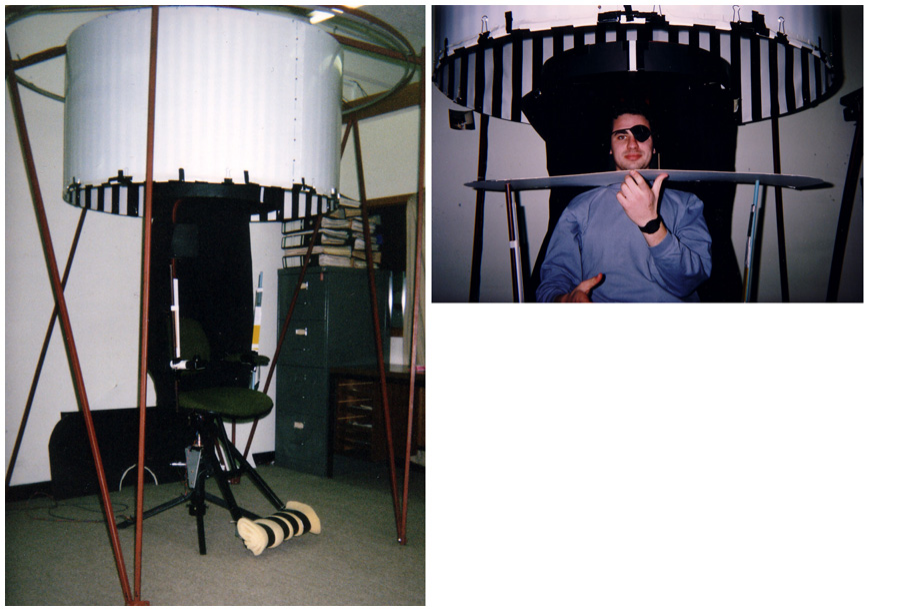
**Rotating-drum-and-chair apparatus used in Palmisano and Gillam (1998).** The drum here is seen in a raised position. During actual testing the drum was lowered so that all the subject could see was the black-and-white stripe pattern on the inner wall of the rotating drum. As in the earlier Brandt et al. (1971) study, illusory self-rotation induced by the rotating drum was subjectively indistinguishable from real chair rotation. In addition to a traditional verbal rating of the speed of self-motion, this study also utilized an objective behavioral measure of vection. Each subject was asked to “set yourself until you feel stationary using the hand-control – when you feel that you are stationary, I’ll get you to tell me.” Unknown to the subject, he/she was physically stationary for the first 40 s of any drum rotation and this setting actually involved speeding the chair up from stationary (in the same direction as the drum motion). Conditions with greater vection-inducing potentials (e.g., full-field, as opposed to 25° diameter, motion stimulation), and which produced faster vection speed ratings, required faster nulling chair speeds. Thus, this nulling chair speed measure would appear to qualify as an objective indicator of vection (discussed in Challenge 3).

Multiple senses contribute to the perception and control of self-motion, including the visual system, the vestibular system of the inner ear, the somatosensory system of cutaneous receptors, the proprioceptive system of muscle and joint receptors, and the auditory system (Dichgans and Brandt, 1978; Howard, 1982). These sensory systems register both the optical (and other sensory) flow produced by the world moving past our head, as well as the pressure/forces applied to our bodies as we move. While the appropriate stimulation of any of these senses can (under favorable circumstances) generate a perception of self-motion, vision appears to play an especially important role. This is convincingly demonstrated by the fact that highly compelling illusions of self-motion can be generated by presenting large patterns of optic flow to physically stationary observers. For example, when seated inside a large rotating drum with an alternating pattern of black and white stripes on its inner wall (see **Figure 1**), individuals typically experience a visually induced illusion of self-rotation that is subjectively indistinguishable from their chair actually rotating (Brandt et al., 1971; Palmisano and Gillam, 1998). Traditionally, such visual illusions of self-motion were referred to as ‘vection.’ However, the term ‘vection’ has, over the years, been used in a variety of other ways (see **Table 1**).

**Table 1:** Data obtained from the top 100 hits in the Web of Science for ‘vection’ as a topic (sorted by ‘relevance’; Publication dates range from 1991 until 2014; Search conducted on November 18, 2014).

Vection definitions	Percentage
#1. Visual illusion of self-motion	52
#2. Illusion of self-motion	15
#3. Visually mediated self-motion	16
#4. Subjective experience of self-motion	1
No definition or ambiguous	10
Could not be sourced	6

**FIGURE 2:**
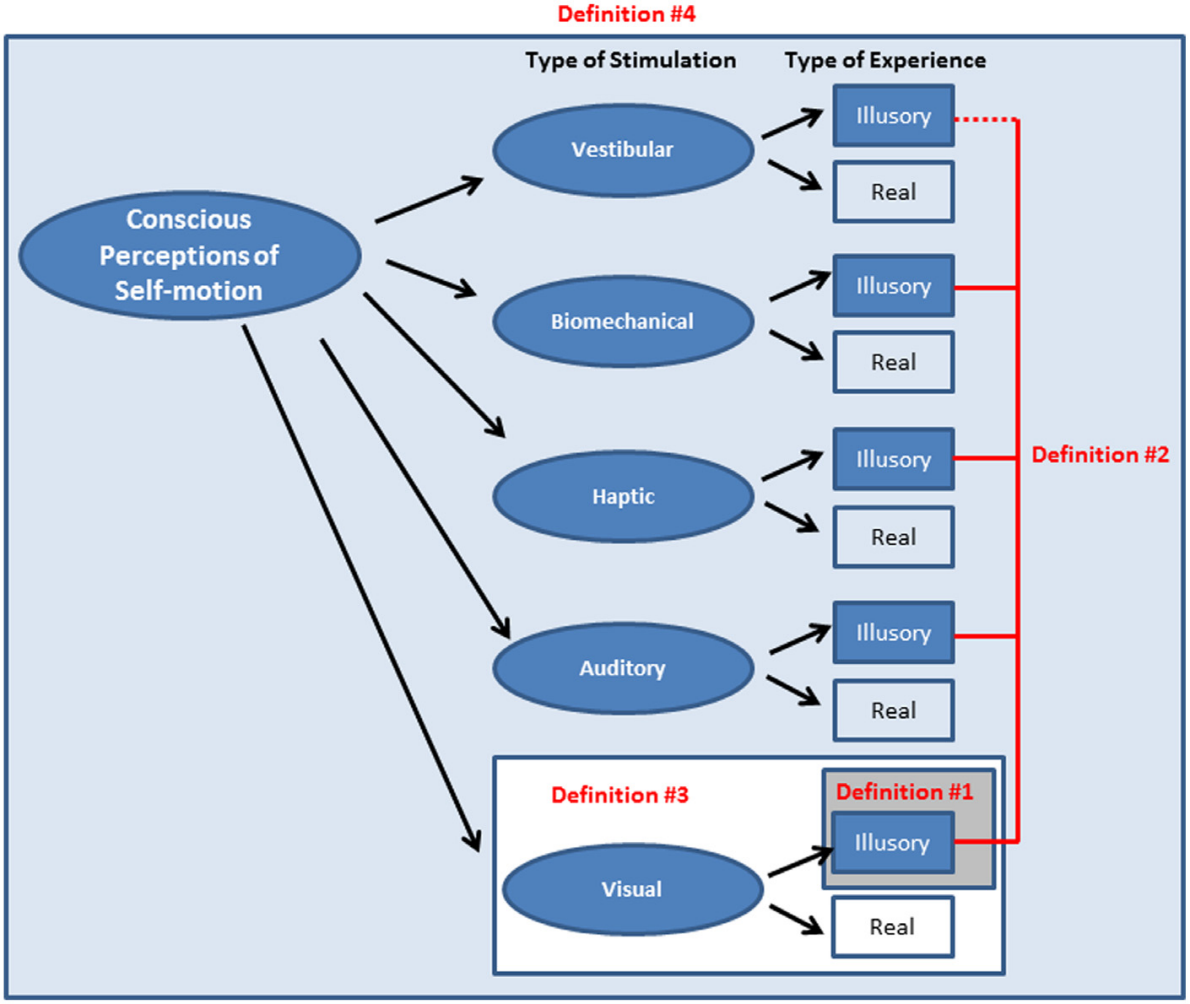
**Various possible definitions of vection.** Shaded boxes show the relative relation between the definitions and also how the scope of the phenomena considered to be vection becomes increasingly broad from definition #1 through to #4. The dotted line for Definition #2 is meant to indicate that while vestibular stimulation can generate illusions of self-motion in physically stationary observers, the term “vestibular vection” is rarely used (see **Table 1** on page 2 for the relative frequency of use of these four definitions).

*The table shows the usage percentages for each of the four definitions of “vection” in these papers (see **Figure 2** on page 4 for a pictorial representation of the relationships between these different definitions).*

This paper discusses four major challenges that we believe face modern vection research. Challenge 1 (Defining Vection) outlines the four main ways that vection has been defined in the literature and discusses their theoretical and experimental ramifications. It is noted that the common thread in all of these definitions is the conscious subjective experience of self-motion. Thus, Challenge 2 (Significance of Vection) tackles the crucial issue of whether such conscious experiences actually serve functional roles during self-motion. Challenge 3 (Vection Measures) next discusses the difficulties with existing subjective self-report measures of vection, and proposes several more objective measures based on recent findings. Finally, Challenge 4 (Neural Basis) reviews neuroimaging research on the neural basis of vection and discusses the hurdles facing such investigations in the future.

## CHALLENGE 1: DEFINING VECTION

Over the years vection has been defined in several different ways. The four main definitions of vection that have been used are as follows:

### Vection definition #1. A visual illusion of self-motion in a stationary observer

The term ‘*vection*’ (or ‘vecktionen’ and later ‘vektion’ in German) has historically been used most often to describe *visual illusions of self-motion in physically stationary observers* (see the review by Dichgans and Brandt, 1978). This is still the most common definition of vection in the literature today (see **Table 1**). ‘Circular vection’ is typically used to describe visual illusions of self-rotation (such as those induced by the rotating drum apparatus described above), whereas ‘linear vection’ is used to describe visual illusions of self-translation^1^. While research has shown that visual-only stimulation can induce highly compelling vection (Dichgans and Brandt, 1978; Riecke, 2010), these visual illusions can often be enhanced by consistent stimulation of the non-visual self-motion senses (e.g., Wong and Frost, 1981; Schulte-Pelkum et al., 2004; Riecke et al., 2009a,b; Berger et al., 2010; Seno et al., 2011b).

### Vection definition #2. An illusion of self-motion

As noted above, vision is not the only sense capable of inducing illusions of self-motion. The etymology of the word vection comes from ‘*vectio’* in Latin (the action noun ‘to carry’ – see OED Online, 2014). Thus, the implication is that the observer is being carried along by their apparent motion. Given that there is no inherent visual connotation in the Latin root of the word, it is perhaps no surprise that ‘vection’ is now increasingly being used to also refer to *illusory self-motions induced by stimulating the non-visual self-motion senses*. These non-visual illusions of self-motion (where the observer is typically either seated in darkness or blindfolded) include: (1) *Auditory vection* – illusory self-motion induced by moving the observer’s acoustic surround (Dodge, 1923; Lackner, 1977; Sakamoto et al., 2004; Riecke et al., 2008; Keshavarz et al., 2014; see Väljamäe, 2009 for a review); (2) *Haptokinetic vection* – illusory self-motion produced by applying tactile motion stimulation to large areas of the observer’s body (Dichgans and Brandt, 1978; Nilsson et al., 2012; Nordahl et al., 2012; Murata et al., 2014); (3) *Arthrokinetic vection* – illusory self-motion induced by passively rotating the observer’s limb/s (Brandt et al., 1977; Howard et al., 1998); and (4) *Biomechanical vection* – illusory self-motion generated when a standing/seated subject repeatedly steps on a treadmill (Bles, 1981; Riecke et al., 2011). Interestingly, while illusions of self-motion can also be induced by caloric (e.g., Fasold et al., 2002) and direct galvanic stimulation (e.g., Cress et al., 1997; Lepecq et al., 2006), such vestibular illusions are rarely referred to as *vestibular vection* (see below for one notable exception)^2^. Even so, traditional vection now often needs to be preceded by ‘visual’ in research papers to discriminate it from the other non-visual types of vection described above. The common factor in both ‘visual’ and ‘non-visual’ vection is still that, irrespective of the source of the self-motion stimulation, the observer’s overall body position in space does not change (even if, in some cases, their individual body parts may be in motion). Consequently, the critical element of the definition is that the perception of whole body self-motion is always an illusion.

### Vection definition #3. A visually mediated perception of self-motion (real or illusory)

Recently researchers have also started to use the term ‘vection’ to describe experiences of self-motion generated by complex multisensory stimulations. Observers in such studies typically viewed computer-generated self-motion displays while their bodies were physically in motion. The physical motions that accompanied these optic flow displays have been passive (i.e., externally generated) whole-body motions (Wright et al., 2005), or active (i.e., self-generated) head motions while seated (Kim and Palmisano, 2008, 2010; Ash et al., 2011a,b; Ash and Palmisano, 2012), active breaststroke body movements while standing (Seno et al., 2013a), or active walking on the spot (Palmisano et al., 2014a) or even on a treadmill (Onimaru et al., 2010; Seno et al., 2011a; Ash et al., 2013; Palmisano et al., 2014a). Using the illusory definitions of ‘vection’ outlined above to describe self-motion perception in these situations appears problematic. While the focus of this research was primarily on the role that vision plays in self-motion perception, the observers were not stationary and often received redundant visual and non-visual stimulation about their self-motions. This ‘moving observer’ research instead suggests a third way that vection could be conceptualized. Vection could be used to refer to *visually mediated perceptions of self-motion* whether paired with physical motion or not. According to this definition, vection can be studied not only during visual-only stimulation conditions (such as a stationary observer viewing a computer-generated self-motion display), but also during real and illusory conditions with multisensory self-motion stimulations (such as treadmill walking while viewing a visual self-motion display, or actually driving on a straight highway at a constant velocity, etc.). Importantly, this definition has the advantage over purely illusion based definitions of consistency and simplicity in some notable cases. For example, when passive observers are moved smoothly and at constant velocity, the sensory stimulation from their vestibular system soon dissipates. With their eyes closed, these observers would from then on receive no stimulation indicating self-motion. Upon opening their eyes the sensory stimulation would be identical to a classical vection stimulus and it seems inconsistent to treat these cases as fundamentally different. Similarly, practically important cases of visually induced self-motion coupled with onset cues (brief transient vestibular stimuli that help prime vection) and washout (vection stimuli coupled with sub-threshold physical motion cues) would still be classified as vection^3^.

### Vection definition #4. A conscious subjective experience of self-motion (real or illusory)

In principle, vection could be defined even more broadly as the *conscious subjective experience of self-motion (as in* Ash et al., 2013). Interestingly, the earliest definitions that we are able to find (Fischer and Wodak, 1924; Fischer, 1928; Fischer and Kornmüller, 1930) appear to use vection (or rather ‘vektionen’) in exactly this way. Fischer and Kornmüller (1930) describe the sensations of self-motion induced by optokinetic stimulation in the following manner: “*One is tempted to call the sensation of motion in the latter case – the fact that this is an illusion can for now be ignored – ‘absolute sensation of motion.’ They are sensations of the movement of one’s own body. … We call these sensations, following a suggestion from A. Tschermak (1928, 1930), “Vektionen” and we distinguish “Cirkular-Vektionen” (CV) and “Linear-Vektionen” (LV)”* (p. 276) [Translated from the original German text]. Interestingly, there was no distinction between real and illusory sensations of self-motion. So this fourth type of vection definition would be synonymous with perceptions of self-motion or feelings of self-motion. However, it is important to make the distinction between such feelings of self-motion and judgments made about one’s heading, egospeed and time-to-contact with environmental objects (i.e., the assumed functional attributes of self-motion). For example, in the past, many researchers assumed that it was useful to study judgments about the direction of self-motion even though their experimental stimulus displays were unlikely to induce any subjective experience of self-motion – referring to the judgments made somewhat misleadingly as ‘heading perceptions.’ Defining vection as a subjective experience of self-motion would therefore be a useful way to discriminate between the few self-motion studies that did, and the many studies that did not, induce (or assess) feelings of self-motion (referred to as ‘vection’ and ‘non-vection’ based self-motion research respectively). This very broad definition of vection would cover all of the existing research on illusory self-motion perception (both ‘visual’ and ‘non-visual’ vection) and more interactive and increasingly multisensory self-motion stimulation scenarios (‘active’ vection as opposed to ‘stationary’ and/or ‘passive’ vection). Like definition #3 above, this definition of vection would refer to subjective experiences during real and illusory self-motions alike (i.e., ‘real’ and ‘illusory’ vection).

***Summary and implications for challenge 1*.** Vection could in principle be defined: (#1) as a visual illusion of self-motion induced in a stationary observer; (#2) as an illusion arising from either visual or non-visual self-motion stimulation; (#3) as a visually mediated subjective experience of self-motion; or (#4) as any subjective experience of self-motion (see **Figure 2**). How vection is defined has important implications and consequences. Defining vection as an illusion (Definitions #1 and #2 above) might imply to some that it is an unusual error or mistake. Since humans are normally remarkably skilled at controlling their self-motions, these two types of definitions raise the possibility that vection may have little or no behavioral relevance. By contrast, if one instead defines vection as a conscious, subjective experience of self-motion (Definitions #3 and #4 above), then this opens up the possibility that vection might instead be a part of the critical processing involved in perceiving and controlling self-motion. Illusory vection is likely to share mechanisms with real self-motion (although to what degree this is actually the case is currently unknown). This crucial issue of the behavioral relevance of vection is discussed in detail below as part of Challenge 2. For the remainder of this review paper, definitions #3 and #4 of vection are generally favored (as they are broader and more inclusive than definitions #1 and #2). It should be assumed that when vection is mentioned we are talking about a conscious experience of self-motion (unless otherwise specified).

## CHALLENGE 2: DETERMINING THE FUNCTIONAL SIGNIFICANCE OF VECTION

As we travel through the world (e.g., walking or sitting on a moving bus), we are almost always conscious of our own self-motion^4^. This conscious perception of self-motion typically has both a definite direction^5^ (i.e., ‘perceived heading’) and speed (i.e., ‘egospeed’ or ‘perceived speed of self-motion’). We also feel aware of these attributes and of when we will reach targets and collide with obstacles based on our vection (i.e., ‘perceived time-to-contact’). But why are we conscious of our own self-motion? Are these feelings of self-motion necessary? Do they provide any functional benefits in terms of judging and controlling our self-motions? What roles do they play in navigation and spatial orientation? Modern theorists and researchers generally fall into two opposing camps – one which appears to assume that vection is irrelevant in simulation based self-motion experiments, the other maintaining that these conscious experiences actually play important roles in self-motion. The backgrounds and the evidence for each of these positions are discussed below.

### IS VECTION AN EPIPHENOMENON?

As noted above, vection has traditionally been defined as an illusion of self-motion. This implies that vection is an unusual error or mistake, which stands in stark contrast to our ability to accurately control self-motion. Along similar lines, Warren (1995) has noted that while accurate heading judgments can be obtained after only 300 ms exposure to optic flow which simulates self-motion, it typically takes more than 1 s exposure to this flow to induce an illusory percept of vection (generally between 1 and 10 s; Dichgans and Brandt, 1978). Based on these definitions and findings, many researchers appear to have assumed that vection has little or no behavioral relevance^6^. One could argue (for example) that conscious perceptions of self-motion simply take too long to generate, and are too error prone, to be particularly useful.

Successful locomotion is thought to depend on the ability to direct and control self-motions toward goals while avoiding obstacles and maintaining balance. It has long been assumed that the rapid and automatic (i.e., preconscious) pickup of optic flow based information – about our heading and our time-to-contact with objects in the surrounding environment – underlies these behaviors. For example, Gibson (1950) famously and influentially proposed that each of the functional attributes of our self-motion is specified by a single invariant property of the optic flow (i.e., one-to-one mapping). For example, since the focus of expansion (i.e., the point of zero velocity) in the optic flow always lies in the direction of self-motion, he argued that this property was used to judge and control our heading (Gibson et al., 1955). According to Gibson’s theory of ‘direct perception,’ such perceptions and their resulting actions are both rapid and automatic, because they do not require any kind of synthesis or set of inferences.

Along similar lines, supporters of the two-streams hypothesis generally argue: (a) there are two distinct visual processing streams, a ventral stream responsible for conscious perception and a dorsal stream responsible for action (Goodale and Milner, 1992); and (b) “Gibsonian pickup of information is carried out without consciousness by the dorsal system, and … the conscious awareness of certain dorsal system processes is an after-the-fact epiphenomenon resulting from the transfer of the information to the ventral system for registration or assistance when needed” (Norman, 2002, p.90). Thus, these researchers and theorists might argue that our conscious experiences of self-motion are simply intriguing epiphenomena – that is, delayed by-products of the brain activity actually responsible for controlling self-motion.

### IS VECTION FUNCTIONALLY SIGNIFICANT?

There are, however, reasons to question the ‘vection is an epiphenomenon’ argument. First, researchers might have assumed that vection has little or no behavioral relevance because it has been defined as an illusion of self-motion. However, as was noted above, such illusions of self-motion can be thought of as just one example of vection. Rather than being unusual, visually mediated and other subjective experiences of self-motion accompany most self-motions (irrespective of whether they are real or illusory in their origin). Second, while some theorists and researchers point to long vection latencies as evidence that vection has little behavioral relevance, it is important to note that such delays are only found when self-motion simulations generate sensory conflict. Vection (both real and illusory) can be virtually instantaneous when the available multisensory information is consistent with simulated self-motion (e.g., Berger et al., 2010; Ash et al., 2011a) or when the visual stimulus is compelling and enveloping such as a full-scale moving room (Allison et al., 1999)^7^. Finally, while the original support for the two-streams hypothesis came from findings that visual illusions appeared to distort conscious perceptions but not actions (e.g., Goodale et al., 1991), those findings are still somewhat controversial. Several more recent studies have reported that both conscious perceptions and actions can be fooled by such illusions (e.g., perceiving or grasping the central disks of an Ebbinghaus figure – Franz et al., 2000; see Cardoso-Leite and Gorea, 2010 for a recent review of this literature). Thus it is possible that our conscious experiences actually play important functional roles in the perception, control, navigation, or guidance of self-motion. The available evidence about whether vection does indeed play such functional roles is outlined below.

#### Possible functional role #1. Role of vection in making judgments of self-motion

“In principle, one could steer simply on the basis of information about current heading, time-to-contact and the boundaries of objects” (Warren, 1995, p. 308). Based on this logic, most self-motion research has focused on the accuracy/precision of visual heading and time-to-contact judgments. In these traditional psychophysical experiments, stationary subjects typically viewed large numbers of briefly presented, schematic motion displays and responded to each in turn in a forced-choice fashion. For example, in Warren et al. (1988) subjects were asked to judge whether their visually simulated heading was to the left or right of a probe at the end of each briefly presented motion display (28 cm high by 38 cm wide; viewing distance 45 cm). No attempt was made in this or in most other such studies of this type to check for subjective experiences of self-motion (i.e., illusory vection). In fact, the simulations in these studies were generally too small (e.g., presented only on a computer monitor such as the above), too brief (e.g., 0.3–3.7 s) or too schematic (e.g., only a few moving dots) to induce much (or any) vection. Given these artificial conditions, passive tasks, and the likely absence of vection, it is possible that subjects were forced to imagine that they were moving in order to respond properly in these experiments – which if true, would appear incompatible with the measurement of Gibsonian type self-motion perception. Failures of imagination (rather than perception) could explain why it was not uncommon for subjects in such experiments to display left–right reversed heading judgments when they did not experience any vection during simulated translation (or display up–down reversed heading judgments during simulated landing – Palmisano and Gillam, 2005)^8^. Thus, Ito and Shibata (2005, p. 401) recently called for “detailed research comparing vection directions and perceived heading conducted with the same kind of stimuli.” In fact, there are already findings which suggest that performance on this sort of heading task does depend on whether the stimulus conditions are favorable for vection induction or not. For example, Grigo and Lappe (1998) reported that the accuracy of their subjects’ heading judgments changed markedly as the optic flow duration decreased from 3.2 s to only 0.4 s (Note: the latter duration would be too brief to induce any visual illusion of self-motion in a physically stationary observer). Similarly, adding vection-inducing components to self-motion displays has also been shown to significantly alter both reported egospeed sensations (Kawashima et al., 2011) and time-to-contact judgments (Gray and Regan, 2000).

#### Possible functional role #2. Role of vection in controlling self-motion

**FIGURE 3:**
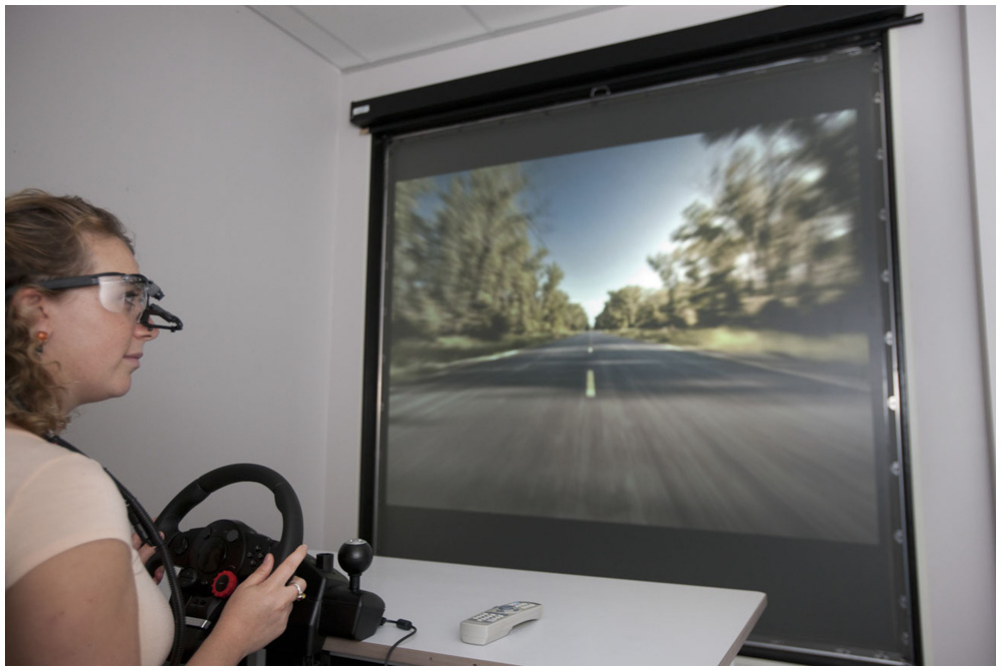
**Simulated driving study.** Interactive self-motion experiments (such as simulated driving studies) rarely checked for subjective experiences of self-motion. The few that have done this reported only whether illusory vection was experienced at some time during the experiment. An example of one such a study is depicted here ( Vinnikov et al., 2013).

While recent simulation studies on self-motion control have generally used more ecological stimuli and experimental tasks (e.g., **Figure 3**), the conditions in most were still not optimal for vection – displays were either too short (10 s or less) or too sparse/basic. Again, very few of these studies checked for subjective experiences of self-motion. The few that did only reported whether illusory vection was experienced at some time during the simulation experiment (e.g., Lee et al., 1997), not when in the trial or even on which trial. It has however been reported that heading judgments and steering accuracy are both enhanced by (a) consistent multisensory information about the self-motion (e.g., as opposed to visual-only information – Telford et al., 1995), and (b) allowing active free gaze (e.g., as opposed to static gaze – Wilkie and Wann, 2003). Interestingly, both of these factors have also been shown to enhance vection (e.g., Palmisano and Kim, 2009; Kim and Palmisano, 2010; Ash et al., 2011b)^9^.

#### Possible functional role #3. Role of vection in navigation and spatial orientation

Vection could play functional roles in both navigation and guidance, and also provide useful feedback on the outcomes of self-motion relative to intent. While it is generally difficult to get lost and disoriented when traveling in the real world, it is interesting that most observers quickly become lost when visually navigating through virtual environments (e.g., Klatzky et al., 1998). Extreme errors can be found when even the simplest spatial orientation tasks are examined (such as “pointing to the origin of locomotion”). For example, Riecke (2008) found pointing performance was poor using optic flow displays which simulated simple self-motions (only one simulated turn) but did not induce illusory vection (In fact, surprising left–right reversals in perceived orientation were seen in many cases). Riecke et al. (2012) have, however, shown that performance on similar spatial orientation tasks can be substantially improved by the induction of illusory self-motion (in this case auditory and biomechanical vection). These findings provide the strongest evidence to date for the functional significance of vection. They suggest that the subjective experience of self-motion helps to update internal representations of our position and orientation in the environment (at least when we are in virtual reality; possibly when navigating in the real world as well). Consistent with this notion, path integration is reportedly more accurate during real walking conditions than in virtual conditions providing only visual information (Chance et al., 1998; Kearns et al., 2002; although Waller et al., 2004 suggested that this benefit was small). Chance et al. (1998) argued that poorer performance in visual-only conditions was due to the absence of vection (A small field-of-view helmet-mounted-display was worn in both their real and visually simulated walking conditions). They noted that a larger display would have promoted vection and speculated that this would have led to more accurate path integration during visual-only conditions. Consistent with their supposition, Tan et al. (2004) found that performance on a ‘return to origin’ task was indeed superior for a larger, distant physical self-motion display compared to a smaller, nearby desktop monitor (that subtended the same visual angle as the larger display).

***Summary and implications for challenge 2.*** Research into the functional significance of vection is still in its infancy. However, identifying the role these subjective experiences play in judging, controlling and guiding our self-motions is likely to have important implications for the design and use of all self-motion simulators and virtual environments. We need to determine whether vection inducing simulations result in better outcomes than displays which look like, but do not make you feel that, you are moving. The answer to this question is of great practical importance – as it is not only difficult, but also expensive and time-consuming to create visual simulations that generate compelling subjective experiences of self-motion. While simulation clearly provides some benefits in the absence of vection (e.g., in terms of procedural learning), anecdotal reports indicate that perceptions, control and guidance are often impaired and serious side-effects are known to occur (e.g., disorientation and motion sickness). Systematic research into these costs is long overdue, and is needed to optimize simulation and training outcomes. Research conducted to date already reveals the importance of vection for spatial orientation in virtual environments (e.g., Riecke et al., 2012). The implication of this work is that subjective experiences of self-motion might also be crucial for successful navigation and the prevention of disorientation in the real world. Riecke et al. (2012) have thus proposed that subjective experiences of self-motion are essential for quick, intuitive, and effortless spatial orientation. However, further research is required to determine whether vection also plays a functional role in visually judging and controlling self-motion. If it is found to do so then it may no longer be appropriate in the future to study simulated self-motion without inducing vection.

## CHALLENGE 3: THE NEED FOR OBJECTIVE INDICATORS OF VECTION

Previous research has relied on subjective self-report measures of vection (e.g., pressing or releasing buttons to indicate vection onset/offset, and rating vection strength continuously with a joystick/throttle). While vection is a subjective experience, and hence naturally measured by subjective responses, these self-report measures are by themselves far from ideal. Self-reported vection onset latencies are likely to be inflated – particularly very early on in an experiment, when the naïve subject first has to decide exactly what constitutes ‘vection’ prior to responding. Self-report measures can also be susceptible to experimenter demands and subject cognitions [such as knowledge about the possibility of actual self-motion and self-motion-bias (as opposed to object-motion-bias) instructions – see Palmisano and Chan, 2004]. One needs to be confident that vection measures are actually capturing the perceived self-motion, rather than the demands of the situation, and are also not confusing any unusual sensations experienced (e.g., feelings of uncertainty or instability, mild symptoms of motion, disorientation or motion sickness) with vection (e.g., see Bonato et al., 2009). Thus, it would clearly be useful to identify alternative/auxiliary indicators of vection, which could be used in conjunction with, and to cross validate, traditional self-report measures.

### POSSIBLE BENEFITS OF OBJECTIVE MEASURES FOR CURRENT VECTION RESEARCH

We feel that there are two main reasons why the development of objective indicators of vection might not only be beneficial, but also quite timely, for modern research:

#### Illusory vection in modern studies is often less compelling

Visual vection has traditionally been induced using very wide field of view stimulation by large physical scene motions. Examples include the rotating drum (Fischer and Kornmüller, 1930; Dichgans and Brandt, 1978; Palmisano and Gillam, 1998), the swinging room (Lishman and Lee, 1973) and the tumbling room (Allison et al., 1999; Palmisano et al., 2006), all of which are capable of generating illusions of self-motion that are subjectively indistinguishable from real self-motion. By contrast, the vast majority of the visual vection research carried out today utilizes smaller field-of-view computer-generated inducing displays. It is widely acknowledged that such displays are less effective vection inducers^10^, which in turn makes them more difficult to study with traditional vection measures. In such situations, these subjective vection measures are more likely to be contaminated by extraneous factors (e.g., experimenter demands and subject expectation/confusion). Unfortunately, many of the visual vection phenomena that are currently of interest can only be practically investigated with computer-generated displays. These include vection without direction (Seno et al., 2012c), vection from second order motion (Gurnsey et al., 1998; Seno and Palmisano, 2011), vection without global motion awareness (Seno et al., 2012b), vection from purely stereoscopic motion (Allison et al., 2014), vection induced by two- and four-stroke apparent motions (Nakamura, 2013), and even vection induced by illusory motion in a flickering stationary image (Seno et al., 2013b). As noted above interest is also increasing into non-visual types of illusory vection, such as auditory, haptic, and biomechanical vection. While these non-visual vection phenomena are both important and intriguing, they are typically much weaker than visually induced illusions of self-motion (even those induced by computer-generated displays – e.g., Väljamäe, 2009). Objective indicators of vection could potentially be of great benefit when examining the weaker (visual and non-visual) vection phenomena described above.

#### Cognitive influences on vection now a major research focus

Since 2000, there has been a dramatic increase in interest in the role that higher-level, top–down, cognitive influences play in vection. Popular topics of research in this area have included the role of stimulus naturalism/realism in illusory vection (e.g., Schulte-Pelkum et al., 2003; Riecke et al., 2005, 2006; Bubka and Bonato, 2010; Riecke and Schulte-Pelkum, 2013), the effect of knowledge about the possibility of actual motion on illusory vection (Lepecq et al., 1995; Palmisano and Chan, 2004; Schulte-Pelkum et al., 2004; Wright et al., 2006; Riecke, 2009), the role of experimental instructions and demands on illusory vection (Palmisano and Chan, 2004; Ogawa and Seno, 2014), the effects of mental imagery on vection (Mast et al., 2001), the effects of stimulus meaning on vection (e.g., figure-ground status and semantic meaning; Seno et al., 2009; Seno and Fukuda, 2011; Ogawa and Seno, 2014) and even the effects of the observer’s own personality characteristics on vection (such as narcissism – Seno et al., 2011d). One intriguing recent study has even reported that vection can be induced solely by cognition (i.e., in the absence of explicit motion – Seno et al., 2012a). The role of top–down cognitive factors on vection is an important area of investigation. However, extra care must be taken when examining these sorts of influences to ensure that subjects are indeed responding to the vection rather than simply the demands of the situation. Objective indicators of vection would therefore be very useful in advancing and validating research in this area.

### CANDIDATES FOR OBJECTIVE INDICATORS OF VECTION

Having identified the need for objective indicators of vection, several possible candidates, and the evidence supporting their potential for use, are discussed below.

#### Possible vection indicator #1: eye-movements

**FIGURE 4:**
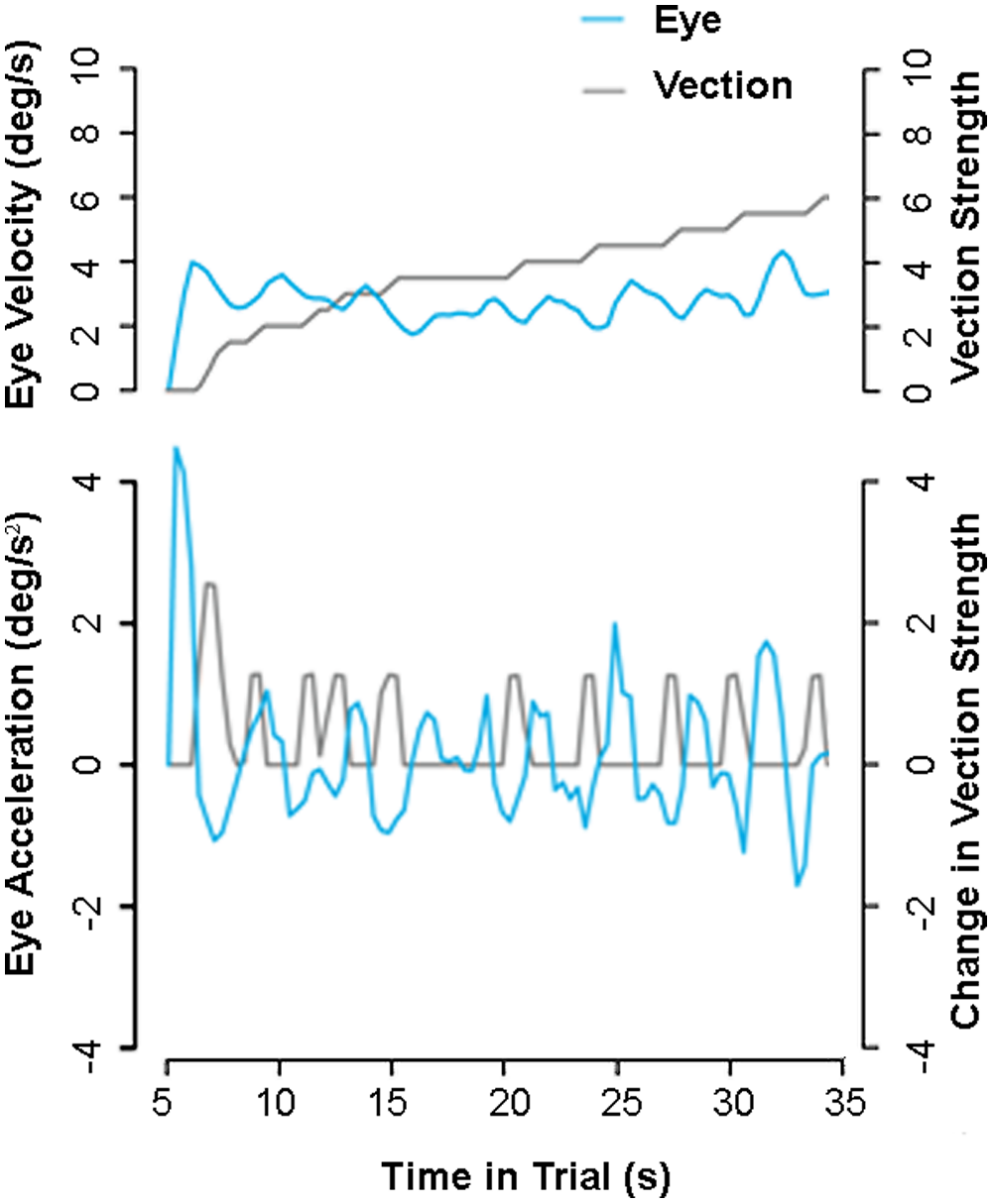
**Possible eye-movement indicator of vection.** One subject’s filtered eye-velocity (above) and acceleration (below) while viewing a 30 s display simulating self-motion over a ground plane. Eye decelerations (cyan lines) appear to predict each of the observer’s subsequent reported increases in vection strength (gray lines). Note this figure presents previously reported data from Kim and Palmisano (2010; Experiment 2).

Compensatory eye-movements made during self-motion consistent optic flow provide one possible candidate for an objective vection indicator. Kim and Palmisano (2010) reported that changes in such eye-movements over time were correlated with reported increases in vection strength. Observers in their experiments were asked to look approximately upward, downward, leftward, or rightward into the 3D cloud or ground plane optic flow for the entire 30-s trial (there was no fixation point or stationary reference). Kim and Palmisano found that increases in instantaneous vection strength ratings were consistently preceded by reductions in slow-phase eye-velocity (see **Figure 4**). The first significant reduction in eye-velocity was typically found to occur just prior to vection onset. Similar reductions in slow-phase eye-velocity can be observed prior to each subsequent increase in vection strength during the 30-s trial. These and similar findings (e.g., Palmisano et al., 2012) suggest that an eye-movement based index of the vection time course should be possible when the observer freely views the self-motion display under certain conditions.

#### Possible vection indicator #2: electroencephalography (EEG)

**FIGURE 5:**
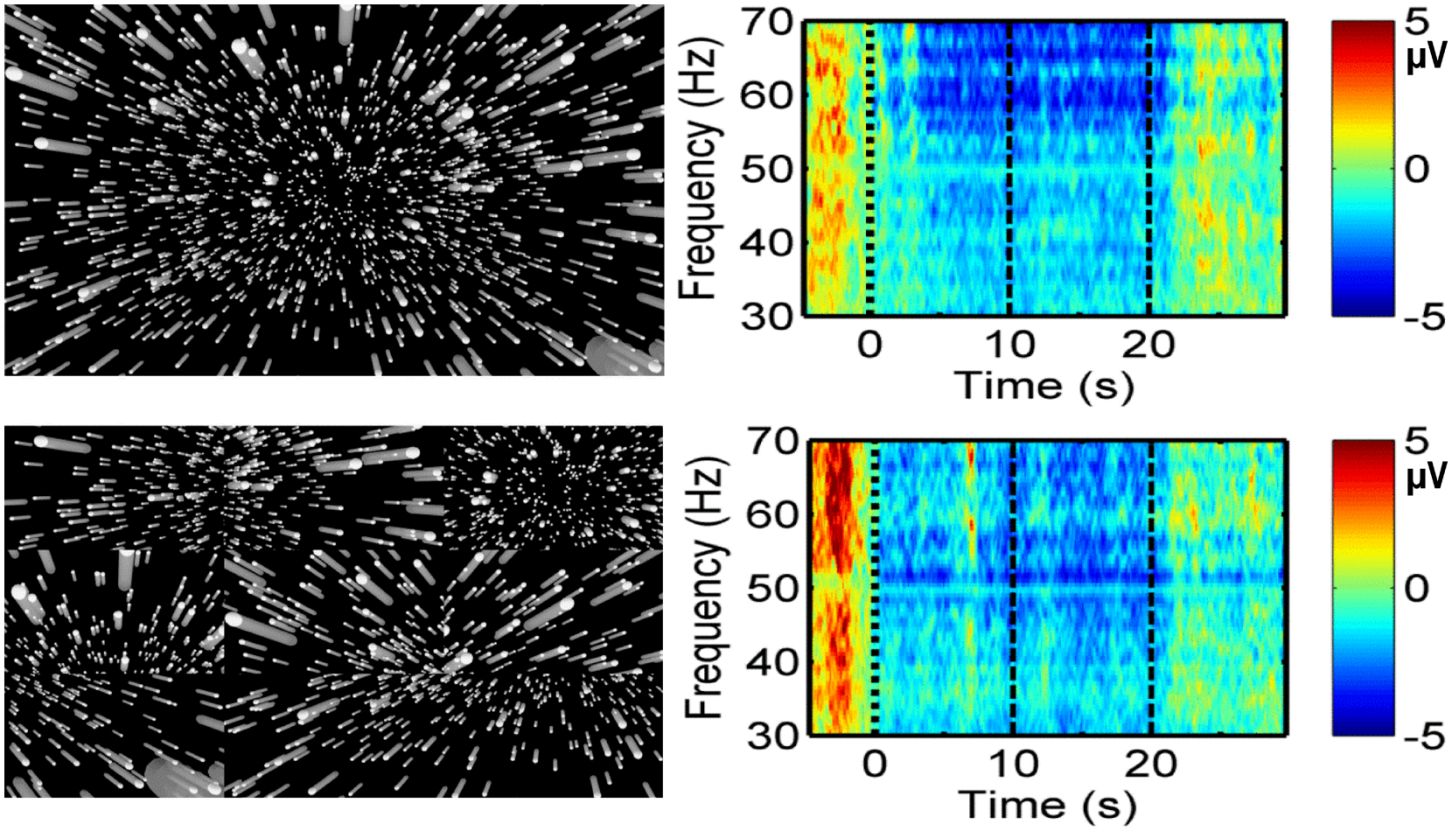
**Possible EEG indicator of vection. Left:** Representations of a radially expanding self-motion-consistent flow (Top) and its spatially scrambled local motion control stimulus (Bottom). **Right:** Corresponding changes in EEG gamma activity at the frontal Fz site in the time-frequency domain (Barry et al., 2014b). Amplitude at each frequency (n) has been scaled by n, yielding the values shown in the voltage scales. Time 0 represents the start of display motion. Display motion continued for 20 s.

Research appears to indicate that electroencephalography (EEG) can be used to discriminate between vection and object-motion perception (Tokumaru et al., 1999; Thilo et al., 2003; Barry et al., 2014b; Keshavarz and Berti, 2014). In the earliest of these studies, Tokumaru et al. (1999) reported significant differences in EEG topography in the high alpha band when circular vection (CV) was induced (compared to viewing the same 120° H × 45° V display when it was stationary). However, these alpha topographic changes showed no common pattern across their five subjects. In a later study, Thilo et al. (2003) measured visual evoked potentials (VEPs) of subjects presented with a large (110° H × 110° V) rotating pattern consisting of black and white radial sectors, which surrounded a smaller stationary checkerboard probe pattern. The black and white squares of the checkerboard pattern changed color every 750 ms. They found the amplitude of the first VEP negative inflection (N70) to the central stimulation was significantly reduced during roll vection (compared to that during object-motion perception) at electrode sites O_Z_ (midline occipital), O_1_ (left occipital), and O_2_ (right occipital). More recently, Keshavarz and Berti (2014) appeared to provide evidence that the N230 at O_1_ and O_2_ may be more pronounced for stronger linear vection (LV) stimuli (although it should be noted that their 2.5–3.5 s displays were too short to induce vection during EEG recording; vection ratings were obtained only with longer presentations of the stimuli after the EEG recording session). Most recently Barry et al. (2014b), using a time-frequency approach to EEG, have compared event-related spectral perturbation (ERSP) data for normal and spatially scrambled vection displays (see **Figure 5**). They found greater event-related desynchronisation in the beta and gamma bands for vection versus scrambled display (control) conditions, and for vection displays which generated stronger vection ratings. These findings support the notion that EEG could provide objective/auxiliary markers of vection.

#### Possible vection indicator #3: postural responses

**FIGURE 6:**
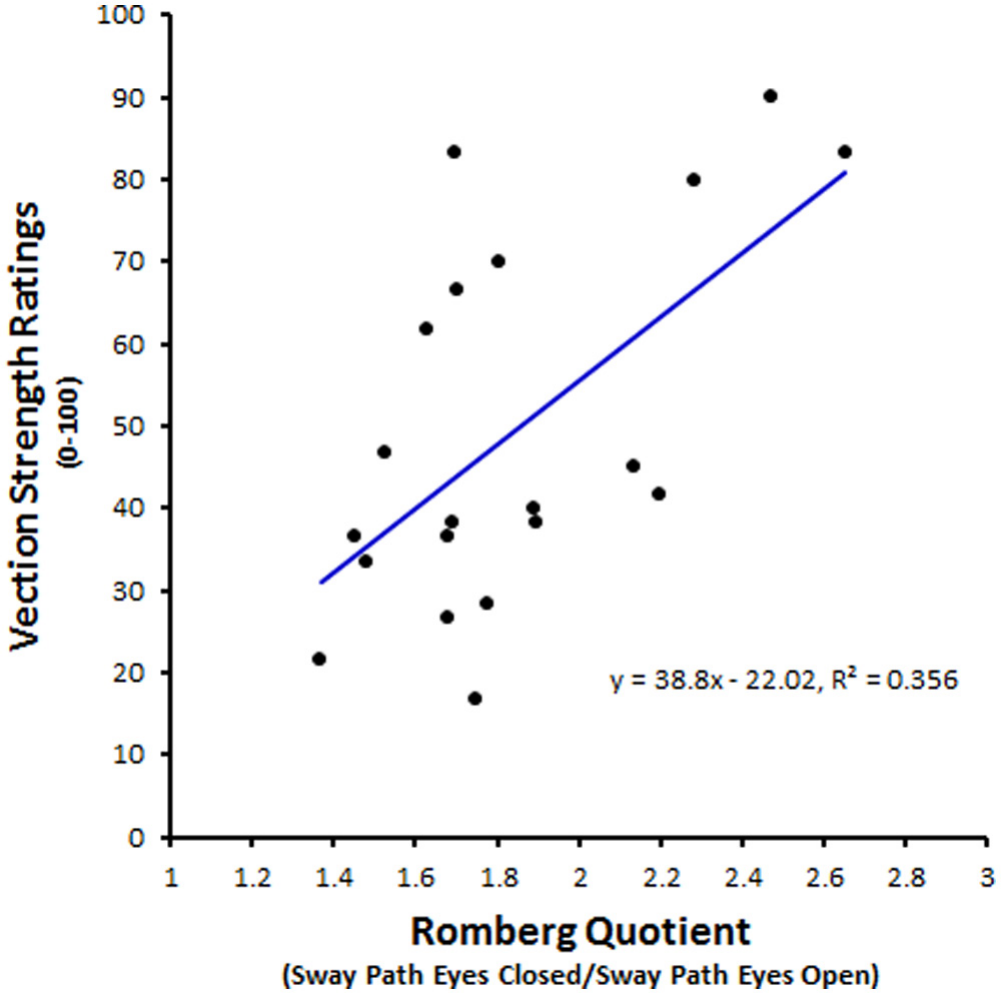
**Possible sway indicator of vection.** This plot shows the relationship between each subject’s Romberg quotient for sway path and their subsequent vection strength ratings (0–100). Path length, a common linear measure used in sway analysis, refers to the distance covered by the standing subject’s center of pressure during the measurement period. Here each subject’s Romberg quotients were calculated by dividing their mean sway path during eyes closed conditions by their mean sway path during eyes open conditions. It should be noted that non-linear analyses of sway data [such as by recurrence quantification analysis (RQA)] appear to be even more predictive of subsequent vection strength than these sorts of traditional linear sway measures (Apthorp et al., 2014). This new figure was created from data reported by Palmisano et al. (2014b).

Postural sway has often been proposed as a promising candidate for an objective indicator of self-motion. Visually induced postural sway can occur without any illusory vection and vice versa (Berthoz et al., 1979; Previc and Mullen, 1990; Warren, 1995). However, visually induced postural sway often has a number of similarities with visual vection – their magnitudes both increase with the area, velocity and spatial frequency of the visual scene motion (Lestienne et al., 1977; Berthoz et al., 1979)^11^. One study by Kuno et al. (1999) reported that increases in visually induced postural sway were followed shortly afterward by increases in vection. Similarly, a number of studies have also reported greater magnitudes of postural sway during vection than during object-motion perception (Thurrell and Bronstein, 2002; Tanahashi et al., 2007; Apthorp et al., 2014). Recent research has even shown that spontaneous postural sway (i.e., prior to any visually simulated self-motion) can be used to successfully predict subsequent vection (Apthorp et al., 2014; Palmisano et al., 2014b – see **Figure 6** for an example).

***Summary and implications for challenge 3.*** Objective indicators of vection appear to be more necessary now than ever – with the increasing use of smaller and often (by necessity) rather contrived computer-generated vection inducing displays, as well as the rapidly growing interest in top–down cognitive influences on vection. In the future, physiological indicators of vection based on eye-movements, EEG and postural responses are likely to provide important confirmatory evidence that what is being investigated is actually the subjective experience of self-motion. Other physiological indicators of vection may also be developed, for example, based on skin conductance, heart rate and pupil diameter changes. However, it is important to note that other objective indicators of vection could also be behavioral in origin (e.g., Palmisano and Gillam’s “nulling chair speed” – see **Figure 1** caption). When given the opportunity to respond to the simulation, observers should behave in a manner that is highly consistent with them actually experiencing the self-motion (e.g., stepping hard on the brake of the driving simulator when the car/truck beside them rolls forward at a traffic light). We might also expect observers to display significantly inflated thresholds for object-motion during certain types of vection (e.g., Probst et al., 1984). Such perceptual and behavioral checks are difficult to design into experiments, but would also provide compelling auxiliary evidence of vection^12^. As noted above, irrespective of its exact definition, vection is always a subjective experience of self-motion. Thus, these proposed objective indicators of vection outlined above would always need to be measured in conjunction with traditional self-report measures of vection (irrespective of whether they were physiological or behavioral in origin). These objective/auxiliary vection measures would validate and confirm, and also in some cases extend our understanding of, the vection data obtained from the traditional subjective self-report measures.

## CHALLENGE 4: UNCOVERING THE NEURAL BASIS OF VECTION

In recent years, functional neuroimaging has become increasingly popular for studying self-motion perception. Most of these studies have investigated the brain activity generated when ‘self-motion-consistent’ visual stimulation is presented to physically stationary observers (e.g., Brandt et al., 1998). Comparatively little research has been conducted into brain activity during vestibular or multisensory self-motion stimulation (e.g., Wenzel et al., 1996; Suzuki et al., 2001; Fasold et al., 2002). While functional magnetic resonance imaging (fMRI) has been employed most frequently in these studies, positron emission tomography (PET) and magnetoencephalography (MEG) have also been used. As it is not currently possible for observers to actually move during such scanning, illusory vection is increasingly being used to generate self-motion perceptions in such studies. While it has only rarely been used to examine the neural basis of self-motion perception (e.g., Tokumaru et al., 1999; Barry et al., 2014b), full-cap multichannel EEG can be recorded during observer motion and has the benefits of a very high temporal resolution. Furthermore, low resolution brain electromagnetic tomography (LORETA) source identification (e.g., Barry et al., 2014a) could be used to localize the brain sources of such EEG based vection markers. Existing neuroimaging research on the processing of self-motion is reviewed briefly below.

### RESEARCH ON THE NEURAL BASIS OF VISUAL SELF-MOTION PERCEPTION

There are an abundance of regions in the human cortex that are sensitive to visual motion. Many lie along the dorsal visual pathway, which starts at the striate cortex (V1), passes through several extrastriate areas such as V3A and MT/V5, before terminating at higher areas of the temporal and parietal lobes. Functional neuroimaging studies have attempted to identify the neural correlates of visual self-motion perception by examining the brain activity generated by ‘self-motion-consistent’ optic flow (e.g., de Jong et al., 1994; Brandt et al., 1998; Tokumaru et al., 1999; Previc et al., 2000; Beer et al., 2002; Kleinschmidt et al., 2002; Deutschländer et al., 2004; Kovács et al., 2008; Wall and Smith, 2008; Wall et al., 2008; Cardin and Smith, 2010, 2011; Pitzalis et al., 2010, 2013; van der Hoorn et al., 2010).

Most of these studies have searched for areas displaying *differential activation* to this globally coherent optic flow versus certain types of control stimuli. Unfortunately, the control stimuli used have varied quite markedly from study to study – some using static dot patterns as controls (e.g., Tokumaru et al., 1999; Deutschländer et al., 2004), others using random/incoherent dot motions (e.g., Cardin and Smith, 2010, 2011), and still others using either spatially scrambled versions of the original self-motion stimulus^13^ (Barry et al., 2014b) or patterns constructed from multiple miniatures of this original stimulus (Wall and Smith, 2008).

Instead of comparing the brain activity generated by self-motion and control displays, an alternative approach has been to examine only self-motion consistent displays and compare brain activity during the periods when illusory vection is and is not experienced (e.g., Brandt et al., 1998; Kleinschmidt et al., 2002; Kovács et al., 2008). While this approach is not without its own difficulties^14^, it does have some important advantages: (a) the experimenter knows if and when vection occurs during scanning (rather than assuming that it always occurs); and (b) the visual motion stimulation during periods of vection and object-motion perception is identical (thereby removing one strong potential confounder that different visual stimuli can introduce).

**FIGURE 7:**
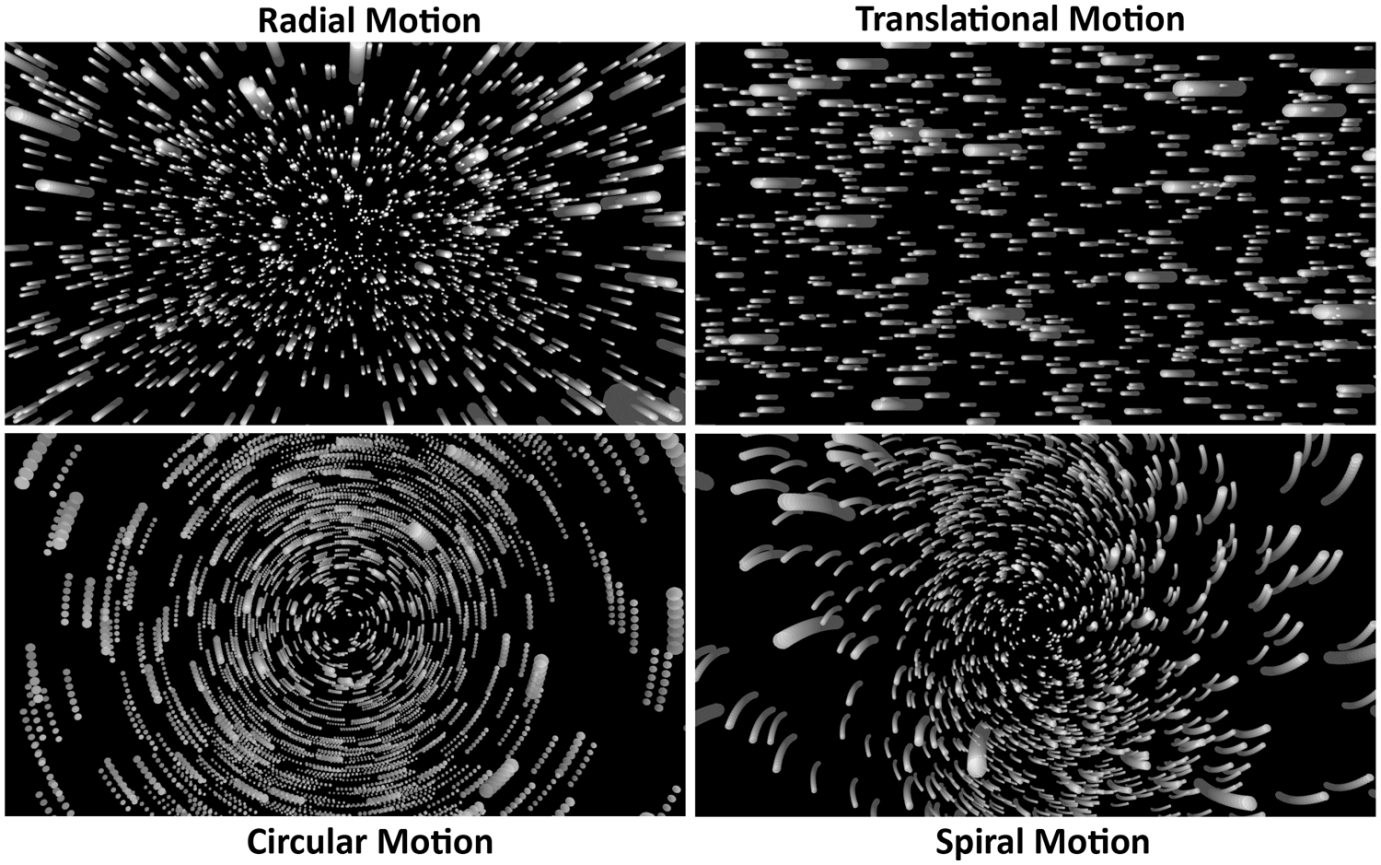
**Representations of four different types of self-motion-consistent optic flow**.

To date, a number of cortical areas have been implicated in the processing of visual self-motion information, including the medial temporal area (MT/V5), the medial superior temporal (MST) area and its dorsal subdivision (MSTd), the dorsomedial area (V6), the cingulate sulcus visual (CSv) area, and the ventral intraparietal (VIP) area. Vestibular/multisensory areas of the cortex have also been implicated, including the intra-parietal sulcus motion (IPSmot) area, the parieto-insular vestibular cortex (PIVC) and putative area 2v (p2v), as well as the precuneus motion area (PcM). Visual self-motion perception may indeed be processed in this highly distributed manner. However, there are also reasons to doubt that the processing involved is quite this complex. First, there has been considerable disagreement and debate about the involvement of several of these brain regions in self-motion processing (such as MST/MSTd – see Morrone et al., 2000; Kleinschmidt et al., 2002; Wall and Smith, 2008). Second, while self-motions can generate many different types of global optic flow (i.e., radial, translational, circular, and spiral patterns of optic flow – see **Figure 7**), most neuroimaging studies have only investigated a single type of self-motion-consistent flow (e.g., radial motion only), and the types of flow examined have varied from study to study (e.g., Brandt et al., 1998; Tokumaru et al., 1999; Wiest et al., 2001; Kleinschmidt et al., 2002; Nishiike et al., 2002; Thilo et al., 2003; Wall and Smith, 2008; van der Hoorn et al., 2010). Third, while differential brain activity for self-motion-consistent and control displays might indeed provide evidence of self-motion processing in some studies, it is possible that such differences might have been generated by irrelevant stimulus differences in others. For example, Tokumaru et al. (1999) found different activation for dynamic self-motion displays compared to that produced by a static control – but is this evidence for cortical self-motion processing? Or does it merely show differences in the processing of moving and stationary versions of the same stimulus? Finally, many studies did not check whether their displays were capable of inducing vection, let alone whether they actually induced vection during scanning (e.g., de Jong et al., 1994; Previc et al., 2000; Wall and Smith, 2008; Wall et al., 2008; Cardin and Smith, 2010, 2011). Of the handful of studies that checked for vection, this was typically done outside the scanner (often using larger displays and/or longer durations than inside the scanner). Thus, with only a few exceptions (e.g., Brandt et al., 1998; Kleinschmidt et al., 2002; Kovács et al., 2008), it is unclear which of the neuroimaging experiments conducted to date actually induced vection during scanning.

### RESEARCH ON VISUAL-VESTIBULAR CORTICAL INTERACTIONS DURING SELF-MOTION PERCEPTION

Unfortunately, the few neuroimaging studies examining human visual-vestibular interactions have generated apparently conflicting results. Early research by Brandt and colleagues found evidence of intriguing reciprocal visual-vestibular cortical interactions during self-motion perception, reporting that: (a) cortical activity in vestibular areas (such as PIVC) was suppressed during vection; and (b) cortical activity in a wide variety of visual areas (including MSTd) was suppressed during vestibular caloric stimulation (Wenzel et al., 1996; Brandt et al., 1998; Deutschländer et al., 2004). These findings suggest that sensory information which is incompatible with self-motion perception is simply suppressed. However, the findings of Nishiike et al. (2002) have complicated this story. Instead of observing vestibular suppression during their accelerating self-motion displays, they reported visual-vestibular co-activation (i.e., activity increased in both MSTd and PIVC, despite the lack of any vestibular end organ stimulation).

In a recent review paper, Palmisano et al. (2011) proposed an explanation of these apparently conflicting findings. Based on the research conducted to date, it was argued that ‘vestibular suppression’ might occur only during constant velocity visual self-motion. Since such self-motions cannot be reliably detected based on vestibular inputs^15^, it would not be surprising if vestibular information was suppressed in favor of the visually induced vection in this specific case. By contrast, visual-vestibular co-activation was reported during accelerating vection. We interpreted this intriguing finding as evidence that the sensory conflicts generated by accelerating self-motion displays might be reduced by “indirect activation of the vestibular cortex.” There are several ways that this indirect vestibular stimulation might be generated in a physically stationary observer (e.g., PIVC might have been activated, via the mid-brain oculomotor pathways, by planned/actual eye-movements triggered by the accelerating optic flow). However, these explanations have yet to be empirically tested.

***Summary and implications for challenge 4.*** Further neuroimaging research is required to reveal how, where, and when, visually and vestibularly perceived self-motion is processed in the brain, and the manner in which the different sensory processes involved interact with each other. Disagreements in the literature are likely to be due to methodological differences between studies – in terms of the criteria used to define and identify self-motion brain areas, as well as the choice of self-motion-consistent and control stimuli. Importantly, evidence about the involvement of particular brain regions in self-motion perception has often been inconclusive because there was no confirmation that illusory vection had been induced in the physically stationary observers (Pitzalis et al., 2013). It is likely then that the successful induction of vection in some studies, and the complete lack of vection in others, may explain the surprisingly common failures to replicate brain activation patterns from one study to the next.

## CONCLUSION

Over the years, vection has been defined either as an illusion of self-motion, or as a visually mediated experience of self-motion, or simply as the subjective experience of self-motion. While the defining criteria for vection have varied quite widely, it is generally expected that self-motion should be perceived both consciously and convincingly in vection studies. However, many modern studies which claim to investigate self-motion fail to meet these criteria. In fact, much of the simulation based research into judgments of self-motion and the control of self-motion has failed to either induce or check for subjective experiences of self-motion. While the researchers involved presumably assumed that vection has little or no behavioral relevance, the functional significance of vection is still an open and largely unexplored question. The answer to this particular question will however, have broad ranging implications not only for the study of self-motion, but also for the use and future development of self-motion simulators. For now, it is encouraging to see that vection convincingly improves spatial orientation in virtual reality.

In the near future vection research is also likely to be enhanced by a range of new, more objective physiological and behavioral measures, based for example, on eye-movements, EEG and postural responses. Such measures will obviously be of great benefit when investigating intriguing but relatively weak vection phenomena (such as auditory vection and directionless visual vection) as well as top–down cognitive influences on vection. These measures will not only be able to provide important confirmatory evidence that traditional self-report measures are actually measuring vection, but in some cases they may also dramatically improve our understanding of vection.

Potentially the most promising development for the future of vection has been the growing belief that illusory vection is necessary to examine brain activity related to self-motion in existing scanners. In order to qualify as a region of interest for visual self-motion perception, we propose that brain regions should be selectively responsive to *all* self-motion-consistent optic flow but *not* to control stimuli containing equivalent local (not global) visual motions. Demonstration of selective responding either to a single self-motion-consistent stimulus, or to self-motion stimuli in the absence of any vection, would not constitute sufficient evidence.

Finally, while most of the neuroimaging research conducted to date has focussed exclusively on the role of vision, self-motion is primarily a multisensory experience. Accordingly, one cannot hope to fully understand self-motion processing by examining the role played by vision in isolation. Thus, it is expected that multisensory aspects of vection will become an increasingly hot topic of research in the future.

## Conflict of Interest Statement

The authors declare that the research was conducted in the absence of any commercial or financial relationships that could be construed as a potential conflict of interest.

## Footnotes

1 Interestingly, Dichgans and Brandt (1978) wrote that: “Fischer and Kornmller, (1930a,b) later gave a more complete description of this illusion which, in reference to Tschermak (1931), was named circular vection (CV) or linear vection (LV), respectively, by these authors” (p. 764). However, we were unable to find any mention of ‘vektion’ or ‘vektionen’ in Tschermak’s book chapter. It would appear that the origins of the term are actually much earlier than this (e.g., Fischer and Wodak use the term ‘Linearvektionen’ in their 1924 paper).

2 In this very early study, Fischer and Wodak (1924, p. 1407) reported that caloric vestibular stimulation had the following consequences: “At the same time peculiar sensations of straight progressive movements occur, so called ‘Linearvektionen’ (LV), finally a not clearly analyzable dizziness. Interestingly, nausea is almost entirely missing – even in very sensitive subjects. Symmetric bilateral warm rinsing, causes upright subjects to perceive backward falling, that is in the opposite direction as rinsing with cold water. Again there is the typical chain of events, including the peculiar sensations” [Translated from the original German text]. This is the earliest use of the term ‘vecktionen’ that we can find and it is noteworthy that it is used to describe a vestibular experience of self-motion.

3 While they are not necessarily ‘vection’ *per se*, other interesting cases arise when traveling enclosed in a ship, plane, or train compartment without windows. In these situations vision will correctly indicate that the observer is not moving relative to the vehicle. However, the motion of the vehicle, and the actual self-motion of the observer who is inside it, must therefore be detected and perceived by the other non-visual senses.

4 The salience of these conscious experiences of self-motion can vary quite markedly. For example, when stopped in a car at a red traffic light, one often experiences a vivid and alarming illusion of self-motion as the car directly adjacent rolls forward. Compare this to a more common, everyday experience of self-motion, such as driving home on a highly familiar route after a long day at work.

5 Note, however, that it appears possible to induce ‘directionless vection’ under certain laboratory conditions (Seno et al., 2012c).

6 Of course, it is also possible that these self-motion researchers did not consider the role of vection in their research for other reasons: (1) they might have been most interested in preconscious processing of sensory inputs; or (2) they might have felt the role of vection went beyond the scope of their study; or (3) they might have feared that introducing vection measures would disrupt the processes under investigation; or (4) they might have had concerns because the community appears to be far from establishing concrete definitions of vection or agreed upon ways to measure “it.” It is also worth noting that the literature on vection has often been focused on qualia and has not been linked to ecological relevance.

7 Visually induced vection is generally more compelling when the motion stimulus is larger and/or contains a high density of moving contrasts (see Dichgans and Brandt, 1978; Riecke, 2010 for reviews).

8 Similar failures in imagination were clearly evident in a recent study comparing pointing behavior during real and imagined walking (Campos et al., 2009).

9 It would appear, however, that active whole body control of the self-motion simulation can (at least in some instances) impair the vection experience (Riecke and Feuereissen, 2012).

10 Unless the vection-inducing potential of these computer displays is enhanced in some way (e.g., by adding stereoscopic, changing-size, or simulated viewpoint jitter cues; see Palmisano, 1996; Palmisano et al., 2000, 2011).

11 Note that when vection is experienced with sway, there can sometimes be differences in both the directions and the magnitudes of these self-motion phenomena (Guerraz and Bronstein, 2008; Palmisano et al., 2009).

12 The occurrence of vection (as opposed to general motion) aftereffects could also provide important confirmatory evidence (e.g., Seno et al., 2010, 2011c). However, these can be difficult to find with computer-generated self-motion displays.

13 The aim of this spatial scrambling was for the control stimuli to provide equivalent local, but not global, visual motion stimulation with respect to the experimental stimuli.

14 Indicating vection onsets/offsets/magnitudes during the scan are difficult due to the potential contamination of brain activity by attentional factors and motor responding. This is particularly problematic in fMRI studies due to their poor temporal resolution.

15 The vestibular system of the inner ear detects only accelerating self-motions (Dichgans and Brandt, 1978; Howard, 1982). So, unlike vision, it cannot discriminate between traveling at a constant linear velocity and remaining stationary.
